# Mechanical Deformation Behavior of Polymer Blend Thin Films

**DOI:** 10.1002/marc.202400736

**Published:** 2024-12-31

**Authors:** Geeta Pokhrel, Hyungyung Jo, Nicholas M. Christ, Hyeyoung Son, John A. Howarter, Chelsea S. Davis

**Affiliations:** ^1^ School of Materials Engineering Purdue University West Lafayette IN 47907 USA; ^2^ Environmental and Ecological Engineering Purdue University West Lafayette IN 47907 USA; ^3^ Department of Mechanical Engineering University of Delaware Newark DE 19716 USA; ^4^ Department of Materials Science and Engineering University of Delaware Newark DE 19716 USA

**Keywords:** elastic recovery, in situ mechanics, polymer blend, pseudo‐bicontinuous morphology, thin films

## Abstract

Examining the mechanical properties of polymer thin films is crucial for high‐performance applications such as displays, coatings, sensors, and thermal management. It is important to design thin film microstructures that excel in high‐demand situations without compromising mechanical integrity. Here, a polymer blend of polystyrene (PS) and polyisoprene (PI) is used as a model to explore microscale deformation behavior under uniaxial mechanical testing. Six thin film compositions ranging from pure PS to a 4.5:5.5 ratio of PS to PI are fabricated. The thin films are deformed under compression, tension, and cyclic loadings, while monitoring the behavior utilizing a micromechanical stage and optical microscopy. To calculate the plane strain modulus, a strain‐induced elastic buckling instability technique is employed. The results show that as the PI concentration increases, the plane strain modulus of the films decreases while the fracture strain increases. For the 4.5:5.5 ratio of PS to PI with a continuous rubbery PI phase, the thin films show major recoverable mechanical performance. This behavior is attributed to the mechanical strength of glassy PS combined with the strain energy absorption capability of rubbery PI, enabling elastic recovery. These fundamental observations provide valuable insights for designing mechanically resilient thin films for coatings and flexible devices.

## Graphical abstract

Thin films of immiscible polymer blends of glassy polystyrene (PS) and rubbery polyisoprene (PI) form microphase separated structures with morphology ranging from sea‐island to pseudo‐bicontinuous with increasing concentration of PI. Pseudo‐bicontinuous 4.5:5.5 PS/PI films with discontinuous PS and continuous PI exhibit enhanced mechanical resilience when compared to formulations with continuous PS.

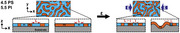

## 1 Introduction

Polymer thin films have been utilized extensively in numerous high‐performance applications, such as energy harvesting technologies, flexible devices, foldable displays, thermal applications, and coatings.^[^ ^1^, ^2^, ^3^, ^4^ ^]^ Thin films used in applications where they are subjected to large strains, in the case of foldable displays for example, must still maintain their functionality irrespective of the extent of deformation. Several different approaches such as nanocomposite and phase separated block copolymer/homopolymer blends have been investigated to enhance mechanical strength while maintaining the desired functionality of the polymer thin films.

Polymer blends made from two or more polymers or copolymers combine the properties of each individual polymer, integrating their characteristics. In immiscible blends, the morphology can vary from sea‐island and fiber‐like structures to lamellar and bicontinuous forms. The morphology of an immiscible polymer blend is sensitive to many factors including composition, molecular weight, film thickness, solvent, rheological properties, and surface properties of substrates.^[^ ^5^, ^6^ ^]^ Bicontinuous morphology of immiscible polymer blends has been reported to optimize toughness, stiffness, and form a percolation path for conductivity in the polymer blend system.^[^ ^7^ ^]^

Besides pure polymer blends, many studies have employed a phase separated system in block copolymers or polymer blends incorporated with nanoparticles to study the bicontinuous morphology of thin films.^[^ ^8^, ^9^, ^10^ ^]^ Using a computational approach, Buxton and coworkers found that the deformation in a bicontinuous copolymer matrix was suppressed by the presence of a percolated rigid network, and the global stiffness of the material was significantly increased.^[^ ^11^ ^]^ Gam and coworkers investigated the morphological evolution in a poly(methyl methacrylate) and poly(styrene‐*ran*‐acrylonitrile) polymer blend films by controlling the thickness of the films and the concentration of inorganic nanoparticles.^[^ ^12^ ^]^ Immiscible polymer blends in which nanoparticles or block copolymers are localized at the phase boundary have been greatly studied because of their unique morphologies with improved properties such as electrical, mechanical, magnetic, and optical properties.^[^ ^13^, ^14^, ^15^, ^16^ ^]^

Previous studies that have analyzed the mechanical properties of polymer blend thin films have mostly utilized tensile tests.^[^ ^17^, ^18^, ^19^ ^]^ However, there have not been sufficient studies to fully understand the mechanical behavior and deformation mechanisms of the polymer blend thin film systems from the initial deformation state all the way to fracture. Additionally, although a bicontinuous or pseudo‐bicontinuous morphology would be promising for stretchable polymer applications, the details of deformation in each morphological phase are not yet completely understood. Rigid polymer components of flexible electronics are often mixed with an elastomer to enhance overall film toughness.^[^ ^20^ ^]^ Our recent study provides detailed observations into the in situ mechanical behavior of thin films of discontinuous glassy and continuous rubbery polymer blends reinforced with boron nitride that form a pseudo‐bicontinuous morphology.^[^ ^21^ ^]^ In that study, boron nitride localized in the continuous rubbery polymer and at the interface of the discontinuous glassy and continuous rubbery polymers.

In the present work, polystyrene (PS) and polyisoprene (PI) polymer blend thin films with various morphologies are investigated to give more fundamental insights into the deformation and failure of thin film polymer blends. We analyze the mechanical behavior of a phase separated thin film fabricated from an immiscible polymer blend. The deformation behavior of each phase is characterized by imaging the thin films during uniaxial compression, tension, and cyclic testing. A micromechanical stage is coupled with an optical microscope to study the deformation behavior. PS and PI are utilized as a model blend with morphologies ranging from cylindrical to pseudo‐bicontinuous, depending on the ratio of PS to PI. The elastic moduli of PS and PI differ by three orders of magnitude, giving representative insight into the interplay between soft and rigid phases in a generalized polymer blend thin films.

Characterizing the mechanical properties of ultra‐thin films of nanometer scale thicknesses can be challenging when using traditional testing methods due to brittleness, handling, and/or measurement sensitivity of the testing apparatus.^[^ ^22^, ^23^ ^]^ To circumvent these challenges, thin films in this study are supported by affixation on a pre‐stretched, elastomeric substrate. During the compressive testing, the stored elastic energy in the soft support substrate transfers to the blend thin films, which enables the observation of subsequent changes on the blend thin film surface as the compressive strain increases. The formation of surface buckles or wrinkles occurs on a thin film adhered on top of a soft elastomeric substrate when the applied deformation exceeds the critical buckling stress of the film. A sinusoidal buckling instability forms on the surface with wavelength and amplitudes dependent on the mechanical properties of the film and the substrate, applied strain, and the thickness of the thin film. A versatile technique, strain‐induced elastic buckling instability of mechanical measurements (SIEBIMM), is applied to determine the plane strain moduli of the blend thin films.^[^ ^24^ ^]^ With this technique, many researchers have studied buckling instabilities including wrinkles, folds, creases, and delamination, and derived the underlying mechanics governing the buckling instabilities.^[^ ^24^, ^25^, ^26^, ^27^ ^]^ SIEBIMM was initially developed and extensively employed for homogeneous homopolymer thin films. However, the technique has since been extended to various composite polymer films including phase separated, sandwich structured, nanoparticle filled, and periodically porous films.^[^ ^28^, ^29^, ^30^, ^31^, ^32^ ^]^ Our study employs the existing understanding of surface buckling instabilities to determine the mechanical properties of the blend thin films with respect to the composition of PS and PI using SIEBIMM. Additionally, this study utilizes cyclic uniaxial mechanical testing to show how the morphology and integrity of the blend thin films are impacted by 100 repeated strain cycles of tension and compression. For all compositions, 10% strain compressive tests and 5% strain 100 cycle cyclic tests were carried out. From these tests, out of all formulations, the 4.5:5.5 PS to PI composition constituting a pseudo‐bicontinuous morphology of PS and PI with a continuous PI phase showed recoverable mechanical performance and was selected for further testing. Tension, compression, and 100 cycles of cyclic testing were performed at 30% strains for the 4.5:5.5 PS to PI thin film. Most of the deformations observed during all tests of the thin films at 4.5:5.5 PS/PI composition were recoverable when returning to 0% strain. During mechanical testing, the continuous rubbery PI phase deforms elastically and the discontinuous PS phase resists deformation to provide resiliency in the thin films.

## 2 Results and Discussion

### 2.1 Morphology of Various PS/PI Blend Thin Films by Composition

**Figure 1:**
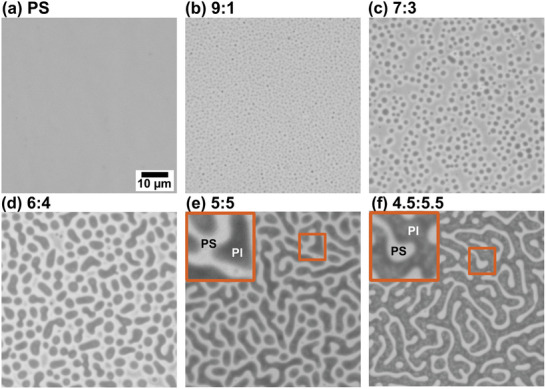
Optical images show morphologies of the PS/PI blend thin films spin coated on a polyacrylic acid (PAA) coated silicon wafer at different PS/PI ratios. The scale bar on the PS image a) applies to all images. Inset images (10 µm x 10 µm) in (e, f) distinguish the PS and PI phases.

Morphologies of the PS/PI blend thin films are shown in **Figure** **1** with respect to the relative weight ratio of PS and PI. The phase separated domains were observed with bright and dark regions, representing PS and PI phases, respectively. As the mass fraction of PI increased, the size of the PI domains increased in a PS matrix until a continuous PI matrix was obtained. Polystyrene‐polyisoprene‐polystyrene (SIS) triblock copolymer compatibilizer, introduced at a constant ratio with respect to the PI concentration, formed isolated islands of PI in 9:1 and 7:3 PS/PI films. At the 6:4 ratio of the PS/PI blend thin film, the PI phase islands had slightly high aspect ratio. A pseudo‐bicontinuous morphology was achieved in the 5:5 blend with a continuous PS phase. In the 4.5:5.5 ratio of the PS/PI blend thin film, the PS phase became the discontinuous domain in a continuous PI matrix. Flory‐Huggins theory provides a theoretical framework in understanding the mixing behavior of polymers and solvents to form stable or phase separated polymer blends based on the free energy of mixing.^[^ ^33^, ^34^ ^]^ In contrast to copolymers having smaller (nanometric) domain sizes, phase separated polymer blends have larger (micrometric) domain sizes. Further, transmission electron microscope (TEM) images show evidence of nanodomains of PS in PI and of PI in PS in a 4.5:5.5 PS/PI film. The diameters range from 100 nm – 500 nm as observed with TEM which are not visible in the optical microscope. Figure S1 (Supporting Information) shows the TEM image for 4.5:5.5 PS/PI representing each phase.

Unlike in melt compounding, the effect of the component polymers’ viscosities is negligible in the spin coating of thin films from dilute solution. The formation of the phase separated domains occurs during spin coating. First, the wetting bilayers of PS and PI are formed on the substrate surface, then fluctuation of the bilayers due to an interfacial instability leads to phase separation. Finally, the morphology is kinetically locked in place as the solvent evaporates.^[^ ^5^ ^]^ During this process, the addition of the SIS triblock copolymer to the PS/PI homopolymer blend acts as a compatibilizer at the interface between the discrete phases of PS and PI.^[^ ^35^ ^]^ Subsequently, the SIS reduces the interfacial tension between the two polymers, leading to a more thermodynamically stable bicontinuous morphology.^[^ ^36^ ^]^ The entanglement between the SIS and the homopolymers also increases the adhesion between the two phases, impacting the mechanical properties of the blend.^[^ ^14^, ^37^ ^]^

**Figure 2:**
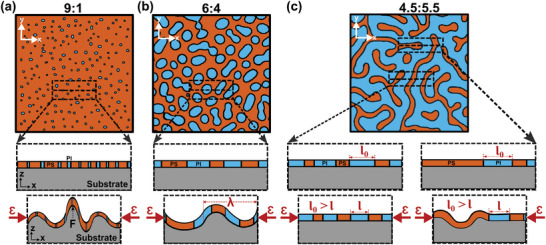
Schematic images illustrating mechanical behavior for 9:1, 6:4, and 4.5:5.5 PS/PI blend thin films under compressive strain. For each composition, the top and middle schematic shows the top view and side view morphologies in unstrained conditions. The bottom schematic for each composition represents the out‐of‐plane buckling instabilities under compressive strain. The extent of out‐of‐plane buckling deformation decreases as the PI concentration in the PS/PI blend increases.

The thickness of the polymer blend thin films ranged from ≈70 to 110 nm as measured with an optical profilometer (OP). The OP was also used to measure the surface roughness of the thin films across different compositions of PS/PI and morphologies, with surface features ranging from 2 to 10 nm. Areal fractions of PS/PI roughly matched the mass ratio of each blend when imaged from the top and bottom of the film. Top and bottom view images also had matching morphology. Therefore, as seen in **Figure** **2**, this study assumes the morphology of the blend is consistent through the thickness of the film, along the z‐direction.

### 2.2 Plane Strain Modulus Calculation via SIEBIMM Technique

Films were measured under a controlled strain condition to characterize reversible and irreversible deformation and to determine the plane strain modulus. The plane strain modulus of the thin films (${\bar{E}}_{f}$) can be calculated using Equation 1 by measuring the wavelength of wrinkles (λ), the thickness of the film (*t*), and the plane strain modulus of the substrate (${\bar{E}}_{s}).$

(1) $${\bar{E}}_{f}=3{\bar{E}}_{s}{\left(\frac{\lambda }{2\pi t}\right)}^{3}$$

Here, the plane strain modulus is defined as,

(2) $$\bar{E}=\frac{E}{\left(1-v^{2}\right)}\;$$

where, *E* is the Young's modulus and ν is the Poisson's ratio.

**Figure 3:**
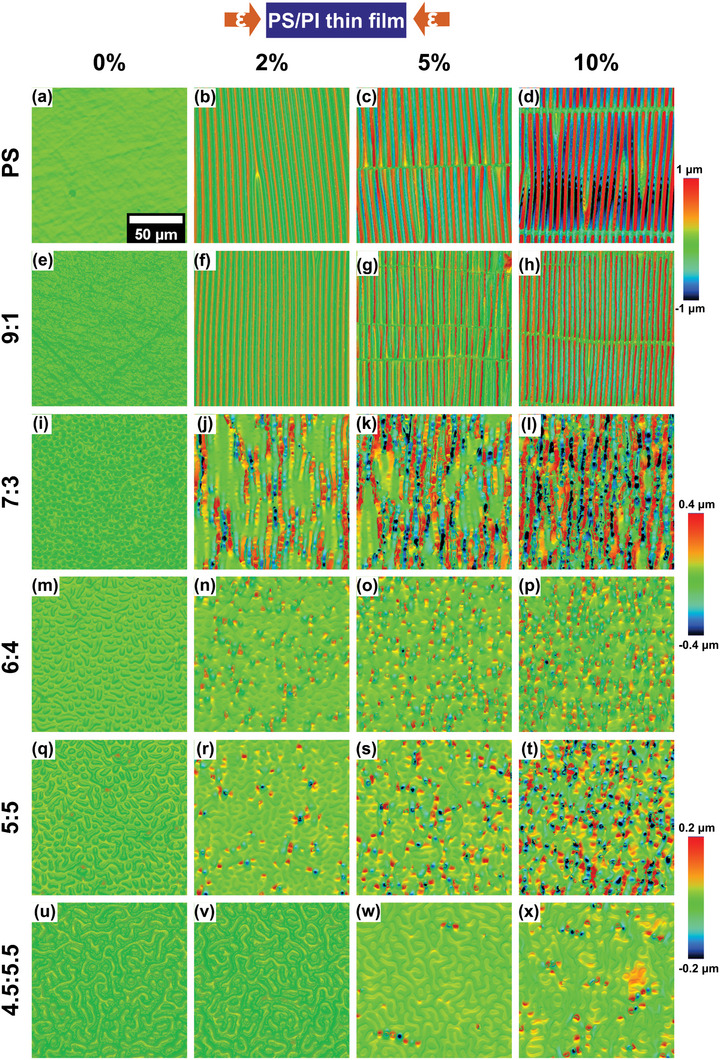
Optical profilometer images show morphology changes of different polymer blend thin film compositions from pure PS to varying ratios of PS/PI on PDMS substrates at ε = 0, 2, 5, and 10% compressive strains. The 50 µm horizontal scalebar in a) applies to all images and the color scale indicates the z‐height for two consecutive formulations of thin film image sets. The height scales for the different thin film formulations are varied to show the details of the out‐of‐plane deformations.

Each PS/PI thin film was transferred and mounted on a prestretched PDMS substrate such that there is 0% strain on the film. When the substrate pretension was slowly released, a compressive strain was applied to the film as the mechanical strain energy stored in the prestretched PDMS substrate transferred to the thin film, inducing out‐of‐plane buckling instabilities. Throughout this work, the reported strain values are strain on the films. Classification of these buckling instabilities depends on test conditions, either stepwise tests as in **Figure** **3** or quasistatic continuous tests as in Figures 6, 7, 8. In stepwise tests, strain was applied with a manual mechanical stage and images were taken at 0, 2, 5, and 10% strains. Quasistatic tests were performed by continuously deforming the films at low strain rates (i.e., 0.0005 s^−1^). The classification of buckling instabilities, especially for pure PS and the 9:1 PS/PI formulations, is discussed in detail in the SI and shown in Figure S2 (Supporting Information).

The extent of deformation of PS/PI polymer blend films in this study varies depending on the concentration of each polymer and the resultant morphologies. The schematic in Figure 2 shows the dominant buckling instabilities for three representative formulations (9:1, 6:4, and 4.5:5.5 ratio of PS/PI) under compressive strain. The middle and bottom schematics represent the side views while unstrained and under compressive strain, respectively. In a quasistatic test, folds are dominant in the 9:1 PS/PI formulation. Out‐of‐plane buckling of the blend thin films decreases as the PI concentration increases. Therefore, bucking instabilities are less severe in 6:4 PS/PI ratio and further reduces in 4.5:5.5 PS/PI due to the continuous rubbery PI that accommodates in‐plane deformations.

Figure 3 illustrates the compressive deformation of all compositions of thin films ranging from pure PS to a 4.5:5.5 ratio of PS and PI as observed with an OP in a stepwise compressive test. The PS and the 9:1 blend thin films developed aligned wrinkles at a 2% compressive strain as shown in Figure 3b,f). At 5% compressive strain, cracks were observed parallel to the compression direction for both PS and 9:1 film because an orthogonal tensile stress was induced due to Poisson effects of the substrate (ν_substrate_ = 0.49).

As the PI concentration increased, the PI domain sizes also increased (i.e., the 6:4 blend thin film PI domains are larger than the 7:3 blend thin film PI domains) as shown in Figure 3i,m). While the 9:1 blend thin film exhibited wrinkling across the entire film at a critical strain, the 7:3 and 6:4 blend thin films partially showed the onset of wrinkling at low strains in the PS domains where the film modulus was locally higher than in the PI domains. The 7:3 film displayed relatively small PI domains compared to the wavelength of wrinkles, and it resulted in lines of contiguous buckling instabilities orthogonal to the compression direction. The PI domains in the 6:4 film showed an in‐plane deformation and there is onset of out‐of‐plane buckling for the PS domain at 2% strain (Figure 3n. As the compressive strain further increased to 10%, the PS continuous phase displayed additional out‐of‐plane buckling which induces out‐of‐plane buckling for the PI domain. The slight increase in PI concentration from the 7:3 to the 6:4 blend led to higher aspect ratio PI domains which were more effective at interrupting the propagation of wrinkles in the PS phase during compression.

Both the 5:5 and 4.5:5.5 films exhibited a pseudo‐bicontinuous morphology. At first glance, both morphologies look perfectly bicontinuous. However, the 5:5 film contains discontinuous PI domains in a continuous PS phase, while the 4.5:5.5 blend thin film has discontinuous PS domains in a continuous PI phase. At 2% strain, localized wrinkling was initiated in the 5:5 film, whereas the 4.5:5.5 film did not show any significant out‐of‐plane deformation. Similar to the 6:4 film, lines of contiguous out‐of‐plane buckling were observed in the 5:5 film at 10% strain. This is because the continuous PS phase buckled, though the high aspect ratio PI domains could partially accommodate in‐plane deformation. On the other hand, the extent of buckling within the 4.5:5.5 blend thin film is significantly lower at 10% strain. At 5% strain in the 4:5:5.5 film, some stress localized regions were observed with isolated buckles occurring on relatively long PS domains aligned along the compressive direction. The length of these high aspect ratio PS domains that buckled upon compression measured ≈10 – 40 µm as shown in Figure S3 (Supporting Information). At 10% strain, most PS domains appeared to rotate to align orthogonal to the compressive direction, as the surrounding PI phase deformed. High aspect ratio PS domains aligned in the compressive direction showed localized buckling instabilities.

In this study, the plane strain modulus was analyzed because the thickness of the thin films is negligible (≈100 nm) relative to the x‐y dimensions of the thin films (≈2×2 mm). For the 4.5:5.5 thin films, it was difficult to measure the representative plane‐strain modulus since wrinkling on the film appeared only on a few small areas of the horizontally aligned PS domains. Thus, the data for the 4.5:5.5 film indicates the localized plane‐strain modulus measured from the partial buckling that occurred on specifically aligned PS domains. Thus, the reported modulus for 4.5:5.5 films was artificially high, skewed by preferentially measuring these aligned PS regions which are much stiffer than the unwrinkled PI regions.

**Figure 4:**
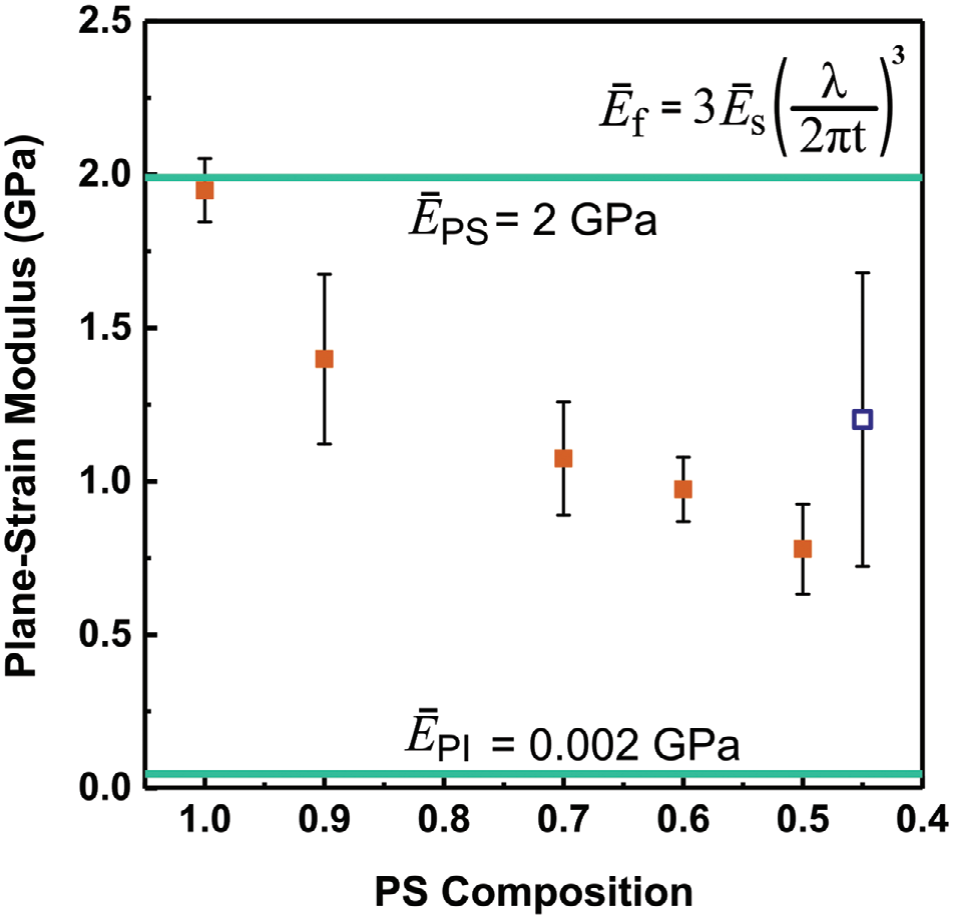
Plane strain film modulus with respect to the PS composition from 1.0 to 0.45. The orange points represent the average plane strain modulus of the PS/PI thin films. For 4.5:5.5 PS/PI thin films, the plane strain modulus, was measured on the PS domains where local wrinkling was observed at 10% strain (represented by the open blue square). The horizontal green lines represent the plane strain modulus of pure PS and PI.

Overall, as the PI concentration increased, the plane strain modulus of the polymer blend film decreased as shown in the graph in **Figure** **4**. Out‐of‐plane buckling instabilities diminished with increasing concentration of the rubbery PI domains due to their ability to deform elastically in‐plane under compressive strain.

### 2.3 Thin Film Deformations under Compression, Tension and Cyclic Strains

**Figure 5:**
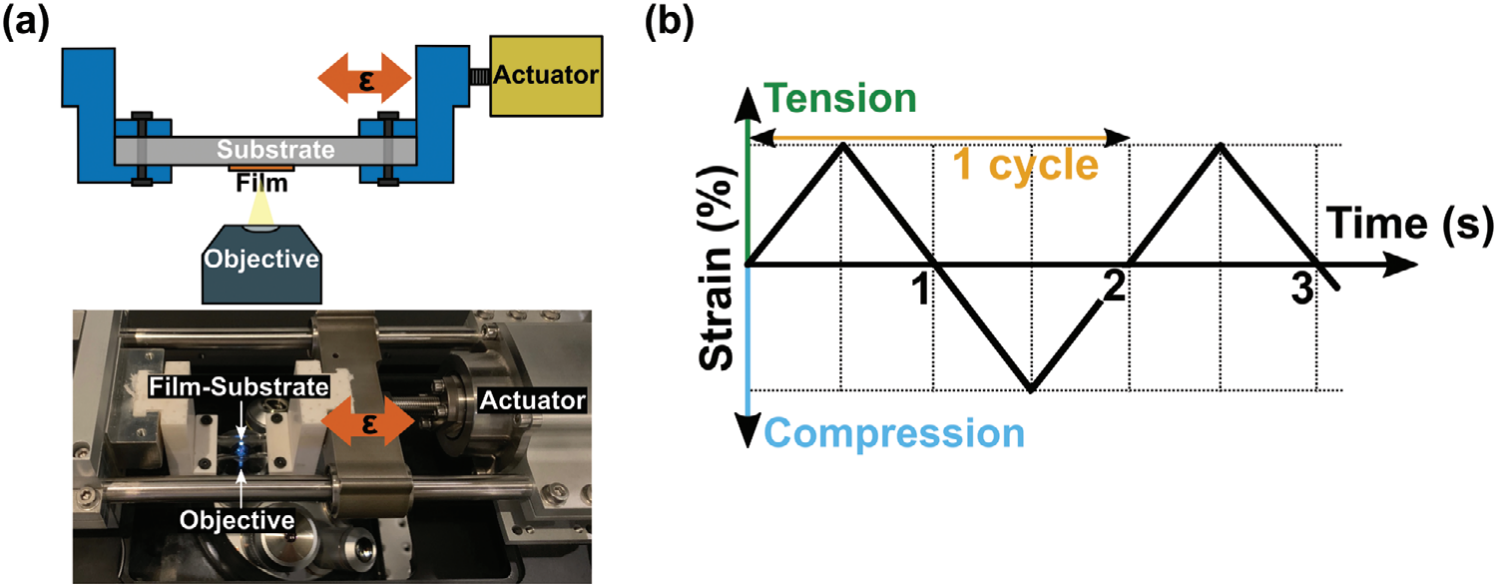
a) Schematic sideview (top) and photograph (bottom) of the in situ mechanical testing setup. The orange arrows indicate the tensile and compressive strain directions. b) Schematic graph of the tension‐compression cycling test protocol with time. One cycle includes one compressive strain and one tensile strain at a particular maximum strain (one and a half cycles shown here). A total of 100 cycles are performed on each sample. The numbers 1, 2, and 3 indicate 0% strain on returning from a particular strain of tension, compression, and tension tests respectively.

The thin films formulated at various PS/PI compositions were deformed under compressive and tensile‐compressive cyclic strains to understand how their morphology affected their deformation during loading. Each thin film composition responds to various mechanical strains differently due to the interaction of glassy PS and rubbery PI. The thin films were deformed under quasistatic, continuously applied strain to monitor their deformation. The 4.5:5.5 PS/PI film was selected as the best candidate composition and evaluated with further compressive, tensile, and cyclic tests. The schematic and photographic images in **Figure** **5**a show the in situ testing setup where a micromechanical stage is coupled with an optical microscope during mechanical testing.

**Figure 6:**
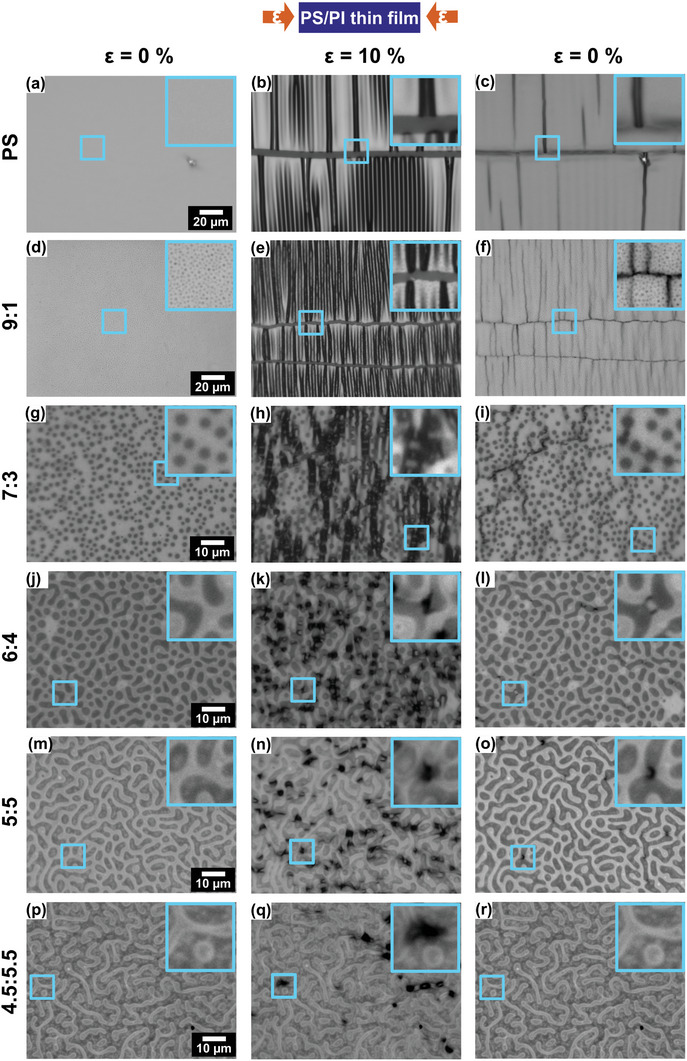
Optical images display morphology changes of PS/PI blend thin films on PDMS substrate at ε = 0% (before test), 10% (maximum strain), and 0% (after removing the strain) in a quasistatic, continuously applied compression test. Inset images show the same area at each strain for the same films. The optical images of the pure PS and 9:1 blend thin film were obtained using a 50x objective, while all others were obtained using a 100x objective. Scale bars shown in the first column apply to their respective rows. SI includes Videos, V1 – V6 (Supporting Information), to show the detailed morphology changes while continuously applying the 10% compressive strain and releasing the strain back to 0% for each thin film formulation.

In‐plane compression of different thin films, from pure PS to 4.5:5.5 PS/PI, resulted in varying combinations of in‐ and out‐of‐plane deformations. **Figure** **6** shows morphology changes of PS/PI blend thin films on PDMS substrate in a quasistatic, continuously applied 10% compression test. Three images were selected from a video of each test: before the test, at 10% compressive strain, and after decreasing the strain back to 0% strain for all compositions of thin films. The Videos V1‐V6 (Supporting Information) are available in the Supporting Information. During compression, wrinkling occurred as the compressive strain was applied to the pure PS film followed by horizontal cracking that occurred rapidly at a threshold strain. Notably, after horizontal cracking occurred on the PS film, further application of compressive strain led to delamination that was initiated from the fractured edges of the cracks. The wrinkling‐to‐delamination transition takes place when the stored strain energy exceeds the adhesion strength of the film‐substrate interface.^[^ ^25^ ^]^ These delaminations remained while the sample was under compressive strain. The delamination width can continue to increase if the debonding energy of the thin film is lower than the energy required to form a new buckle.^[^ ^25^ ^]^ Permanent deformation and fracture occurred on highly stress‐concentrated delaminations though most wrinkles recovered elastically after releasing substrate back to 0% strain, as shown in Figure 6c.

Observation of the wrinkle edges along the horizontal crack showed that further compression of the 9:1 film led to folding at ≈10% strain instead of delamination as shown in Figure 6e. The formation of folds rather than delaminations indicates that a decrease in film modulus due to the introduction of PI has made the wrinkle to delamination transition less favorable. Wrinkling transitioned to folding for the 9:1 blend thin film because the modulus mismatch decreased, while other parameters, the thickness and the adhesion of films, were similarly given in this system.^[^ ^38^ ^]^ After releasing the strain to 0% strain, the folds did not recover, and the film had a higher density of cracking since the 9:1 film had a lower fracture toughness and yield strength compared to the neat PS film.

As the PI concentration increased in the 7:3 film, PI domains were more likely to divert crack propagation in non‐linear and curved ways, while the PS and the 9:1 blend thin film exhibited rapid linear cracking along the lateral direction. As compressive strain was applied, energy dissipation was more likely to occur through the PI phases during crack propagation.^[^ ^39^ ^]^ The 7:3 thin film showed that stress concentrations in PS, along relatively dense lines of PI domains, initiated crack propagation at 10% strain, and the crack remained after returning the strain to 0%. For the 6:4 film, the increased concentration of PI phases could deform more and absorb more strain energy, which led to less localized buckling instabilities in the PS phase compared to the 7:3 film. The inset images in Figure 6i,l show that the buckling resulted in cracking after releasing the strain, while no other significant contiguous cracking occurred along the compressive strain direction.

When the mass ratio reached 5:5, ≈50% of the film area consisted of discontinuous PI phases in the continuous PS phase. At 10% compressive strain, the PS phase showed in‐ and out‐of‐plane deformation by rotating, observed as dark regions located randomly on the PS phase as shown in Figure 6n. These deformations arise from a high variation in local stress due to strain energy absorption by the PI phases. After removing the compressive strain, most of the localized buckling on the 5:5 film was recovered, while some cracks were still prevalent as shown in Figure 6o.

In the 4.5:5.5 film, a unique deformation mechanism was observed that was dependent on the aspect ratio and the orientation of the PS domains in the continuous PI phase. Reduced out‐of‐plane buckling was observed in the PS domains at 10% strain. Since the continuous PI phase has an ability to absorb the strain energy through in‐plane elastic deformation, higher aspect ratio PS domains with their long axis oriented nearly orthogonal to the compressive direction rotated toward the orthogonal direction to accommodate the compressive strain. High aspect ratio PS domains oriented with their long axis nearly parallel to the compressive direction were restricted from rotation. These parallel‐oriented PS domains exhibited out‐of‐plane deformation (periodic surface buckling or wrinkling) to accommodate 10% compressive strain. After releasing the strain, the deformations in the PS domains completely recovered their original appearance, as shown in Figure 6r.

**Figure 7:**
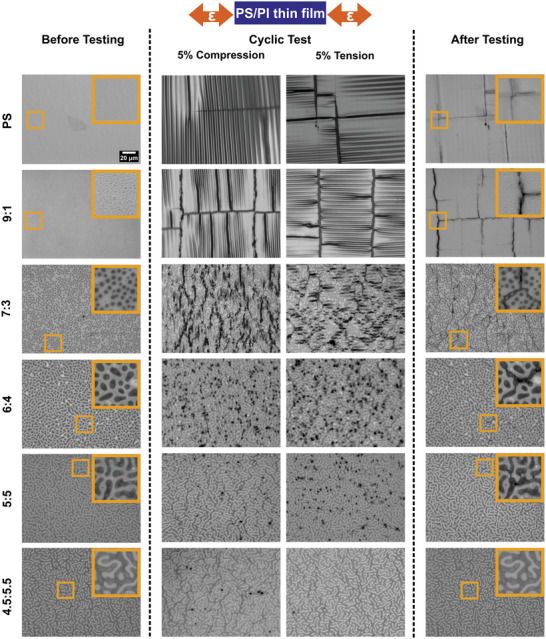
Optical images show morphology changes of PS/PI blend thin films on a PDMS substrate before, during, and after 5% strain compressive‐tensile cyclic testing. The second and third (middle) columns images are 5% compressive and 5% tensile strained morphologies. Images show the same area before and after the cyclic testing. The 20 µm scale bar in the upper left image applies to all large images. Insets (20 µm x 20 µm) show details of the marked positions.

**Figure** **7** shows the morphology of thin films before the test, at 5% compression and tension strains during cyclic loading, and 0% strain after 100 cycles for all six thin film formulations. During the 5% compressive‐tensile cyclic test, a tensile stress was generated orthogonal to the loading direction during compression and a compressive stress was generated orthogonal to the loading direction during the tensile portion of the cycle, due to Poisson effects in the nearly incompressible (ν_substrate_ = 0.49) elastomeric substrate. These opposite, orthogonal stresses are evident through the orientation of aligned wrinkling that occurred at both 5% compressive and tensile strains as shown in the PS and the 9:1 blend thin films in Figure 7. Delamination and crack propagation simultaneously occurred under the cyclic test in the PS thin film.

For the 9:1 blend thin film, crack propagation took place throughout the thin film, both parallel and orthogonal to the tensile‐compressive axis. As the PI concentration increased from 7:3 to 5:5 in the thin films, wrinkling became discontinuous. The PS continuous thin films for 7:3 to 5:5 PS/PI thin films in Figure 7 mainly underwent out‐of‐plane deformation during compressive loadings, indicated by the dark spots shown in the captured images during the cycling tests at 5% compressive and tensile strains.

In contrast to the PS majority films, the 4.5:5.5 blend thin film which had a continuous PI phase, mostly underwent in‐plane deformation except for a few regions where the PS domains had relatively large aspect ratios and were aligned in the compression direction as shown in Figure 7. Cracking was not observed in the PS domains aligned orthogonal to the compressive direction after removing the applied strain. Video V7 (Supporting Information) shows the morphology of a 4.5:5.5 thin film at 0% strain after every 20 cycles of 5% tensile‐compressive cyclic test.

As PI concentration increased from pure PS to 4.5:5.5 PS/PI, Figure S4 (Supporting Information) shows the crack number density changing in two regimes representing crack propagation and crack localization. In pure PS, a low density of long, straight cracks developed over 100 cycles. As more PI is introduced in the 9:1 and 7:3 PS/PI blends, small PI regions cause cracks to branch and increase the overall crack density. In 6:4 PS/PI, the PI domains are large enough to sequester crack initiation and growth, and cracks begin to form directly across small PS bridges between PI regions. From 6:4 to 4.5:5.5 PS/PI, the crack density decreases back to similar crack number density as observed in the pure PS, but the cracks developed by the 4.5:5.5 PS/PI blend film are small and localized.

**Figure 8:**
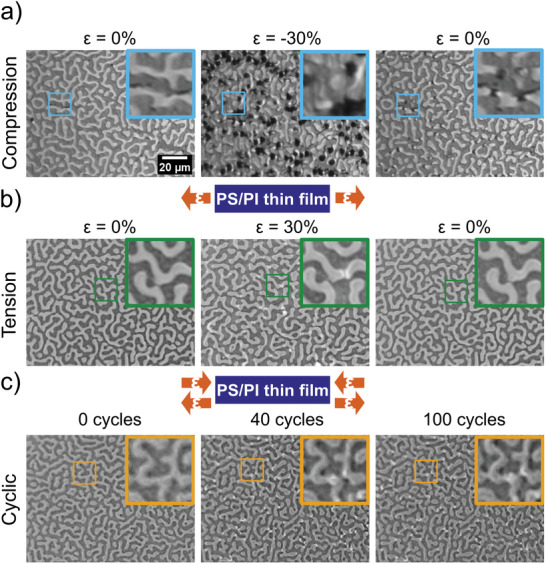
Optical images show morphology changes of 4.5:5.5 PS/PI blend thin films on PDMS substrate before test, at maximum 30% strain, and releasing after 30% strain for a) compression test and b) tension test. c) Morphology at 0% strain at 0 cycle, after 40 cycles, and after 100 cycles of 30% compression‐tension cyclic testing. The scale bar in the upper right image applies to all large images. Inset images (20 µm x 20 µm) show magnified images of the marked positions, representing the same area at different strains or cycle number for a particular mechanical test.

The 4.5:5.5 PS/PI blend was further tested at 30% tensile, compressive, and 100 cycles of tension‐compression cyclic tests to observe mechanical deformation and recoverability as shown in **Figure** **8**. For cyclic tests, images in Figure 8c are shown at 0% strain after 0, 40, and 100 cycles of 30% tensile‐compressive cyclic strains. Videos captured during each mechanical test are shown in SI, V8‐V10. Under 30% compression, a similar out‐of‐plane deformation mechanism was observed as for the 10% strain, but with a higher quantity of deformations. The deformations observed at 30% compression were mostly recoverable. During tension, out‐of‐plane buckling occurred on PS domains oriented orthogonal to the tensile direction. Out‐of‐plane buckling is represented by bright spots in the PS domains. The films fully recovered once the strain was returned to 0% from the 30% tensile strain.

In the 30% cyclic test, localized deformation and damage were accumulated over the course of 100 cycles. Cracks were formed on some large aspect ratio PS domains and localized out‐of‐plane deformations formed on PS domains oriented along the tensile‐compressive axis. The images in the relaxed state after 40 and 100 cycles show major recovery from both the 30% tensile and compressive strains.

The pseudo‐bicontinuous polymer blend thin film of continuous, rubbery PI and discontinuous, glassy PS exhibits an improved mechanical resilience as compared to films with a lower concentration of PI. The 4.5:5.5 film can withstand 30% compressive, tensile, and tensile‐compressive cyclic testing with very little damage, all of which is localized and confined within isolated PS domains.

## 3 Conclusion

By using an immiscible PS/PI blend system, the mechanical behavior of glassy‐rubbery thin films varying in PS/PI composition was investigated. PS and PI blend thin films were fabricated using various ratios of the two polymers to obtain different morphologies of the fabricated films that underwent subsequent mechanical testing and morphological analysis. The pseudo‐bicontinuous phases in the thin films were obtained at a 5:5 ratio of PS/PI constituting a continuous glassy PS phase and in a 4.5:5.5 ratio of PS/PI constituting a continuous rubbery PI phase. Crosslinking of the PI polymer and at the PI and PS interface was achieved by incorporating benzophenone with an SIS block copolymer followed by photo‐activation. This process resulted in improved bonding at the polymer blend interface. The mechanical behavior of the thin films was investigated through in situ uniaxial mechanical testing, and the plane strain moduli were measured using the SIEBIMM technique. When the films had a continuous PS phase, the technique was applicable to measure the effective in‐plane moduli. As the PI concentration increased, the moduli of the blend thin films decreased. PS and the 9:1 PS/PI blend thin films mainly exhibited rapid crack propagation along the compression direction, as loading occurred. However, from the 6:4 PS/PI blend thin film to 4.5:5.5 PS/PI blend thin films, the PI domains in the film prevented cracks from forming due to the dissipative nature of the elastic component.

Above all, a key finding of this study was that the 4.5:5.5 PS/PI blend thin film exhibited a reversible mechanical response up to 30% compressive loading, tensile loading, and 100 cycles of the 30% compressive‐tensile cyclic testing when the blend thin film had a continuous PI phase. The reversible response was attributed to the in‐plane deformation in the continuous PI phase surrounding the PS phase to absorb the strain energy. The localized deformation of the PI phase enabled the PS domains to align normal to the compression direction and prevent any significant failure. In addition, the discontinuous stiff PS phase provides mechanical strength in the blend thin film. Accordingly, a balance in stiffness and elasticity at 4.5:5.5 PS/PI formulation makes the thin films resilient under tension, compression, and cyclic loading conditions at up to 30% strains.

## 4 Experimental Section

### Substrate Preparation

The substrate used in the uniaxial mechanical test was poly(dimethylsiloxane) (PDMS) (Solaris, Smooth‐On, Inc.). A 1:1 by weight ratio of the precursor and crosslinker was mixed, degassed in an evacuated desiccator to remove air bubbles, then poured into 2.5 mm deep glass molds. Next, the mixture was cured for 24 h at 25 °C. For the uniaxial strain test, the PDMS substrates were cut into ≈40×10×2.5 mm rectangular strips. The elastic modulus of the PDMS substrate was measured to be 0.32±0.05 MPa through tensile tests (TA.XtplusC Texture Analyser, Stable Micro Systems) performed at a strain rate of 0.5 mm s^−1^ using ≈20×5×2.5 mm strips.

### Thin Film Preparation

Two polymers, polystyrene (PS) (M_w_ = 93000 g mol^−1^, Polymer Source, Inc.) and *cis*‐polyisoprene (PI) (M_w _ =  35 000 g mol^−1^, Sigma Aldrich), along with a triblock copolymer of polystyrene‐polyisoprene‐polystyrene (SIS) (polystyrene 22 wt.%) were used for the blend thin films. A 6 wt.% solution of polyacrylic acid (PAA) (M_w _ =  102.13 g mol^−1^, Sigma Aldrich) in 70 v/v isopropyl alcohol/water (Fisher Scientific, Inc.) was first spin coated onto cleaned and UV‐ozone (Jelight) treated silicon wafers at 3000 rpm for 30 s as a sacrificial layer to aid in removal and transfer of the blend thin films. Polymer blend thin films were then prepared by spin coating polymer solutions at 3000 rpm for 30 s onto the PAA coated silicon wafers. The total concentration of the polymer blend solution was fixed at 2 wt.% in toluene (Sigma‐Aldrich) and the wt.% ratios of PS to PI were varied between 10:0 (pure PS) and 4.5:5.5 with the addition of 20 wt.% SIS with respect to PI concentration. A constant 5 wt.% of benzophenone (M_w_ = 182.22 g mol^−1^, Sigma Aldrich) to the total amount of polymers was used as a photo‐initiator to crosslink the films. The spin coated thin films were vacuum dried for 20 h at room temperature. They were then crosslinked through benzophenone activation in an oxygen plasma chamber (GLOW Plasma System, Glow Research).^[^ ^40^ ^]^

### Mechanical Deformation Experiments

*Compression Tests at 10% Strain*: The elastomeric substrates were pre‐strained equal to the compressive strain% on a uniaxial micromechanical load frame (µTS, Psylotech). The polymer blend thin films were sectioned roughly into 2 mm by 2 mm strips then floated on water by dissolution of the PAA layer to detach the film from the silicon wafer. The floated film was transferred onto the pre‐strained PDMS substrate using a nichrome loop (Ted Pella, Inc.). At this stage, the blend thin films were assumed to be at ε = 0 before testing. Compressive strain was applied to the thin film by gradually releasing the strain on the pre‐strained substrate as shown in Figure 6a. As the applied compressive strain on the films increased up to 10%, the morphological changes in the blend thin films were continuously observed via optical microscopy (DMi8, Leica Microsystems). Then, the film was returned to 0% strain to observe the reversibility of the deformation. The global strain rate was ≈0.0005 s^−1^. A series of microscopy images were taken at 5 frames per second during testing to generate a video of the surface as the film is being deformed. SI shows Videos V1‐V6 (Supporting Information) taken in a quasistatic 10% compression test and releasing back to 0% strain for all polymer blend thin film compositions. Additionally, the topography of films under compressive strains of ε = 0, 2, 5, and 10% was observed using optical profilometry (NewView 8300, Zygo) to measure the wavelength of wrinkles. This value is needed to calculate the apparent plane strain modulus of the film and to analyze the deformation/failure mechanism of the film. In order to calculate the plane strain moduli of thin films, the wavelengths of wrinkles were measured at strains of 2% for the PS and the 9:1 blend thin film, 5% for the 7:3, 6:4, and 5:5 PS/PI blend thin films, and 10% for the 4.5:5.5 PS/PI thin film using the optical profilometer software (M_x_ Pro_,_ Zygo).

*Cyclic Tests at 5% Strain*: For the cyclic test, the polymer blend thin film was attached to the pre‐strained PDMS substrate in the same way as illustrated above for the compression tests. 5% compressive/tensile cyclic strains were applied on the film‐substrate system for 100 cycles as shown in Figure 6b. The strain rate for 5% cyclic tests was 0.1 s^−1^. In order to observe morphology changes, the compressed/stretched film surfaces were imaged during cycling, and unstrained surfaces before and after testing were also imaged using the optical microscope. A series of microscopic images taken at 0% strain after each cycle are generated as Videos in the V7 (Supporting Information).

*Further Mechanical Testing for 4.5:5.5 PS/PI Blend Thin Film*: For compositions between 10:0 and 5:5 ratios of PS and PI, 10% compressive tests were performed. A composition of 4.5:5.5 PS/PI was selected as the best candidate for a resilient thin film, and further testing was performed. Compression, tension, and cyclic testing were performed at 30% strains for the 4.5:5.5 PS/PI composition. Tension tests were performed only for 4.5:5.5 PS/PI formulation to further study the resiliency it showed in compression. PDMS substrates were pre‐strained by 10% strain before transferring the thin film. To observe the morphology changes during the tension test, the film surfaces were imaged before the test, at maximum strain, and decreasing the strain back to 0% strain using the optical microscope. A series of microscopic images were taken at small intervals ranging from 1.0‐1.5% strain to generate the videos as shown in the Videos V8‐V10 (Supporting Information).

*Optical Images Processing*: Images and videos obtained from optical microscopy were processed using image analysis software (Fiji, ImageJ, National Institutes of Health).^[^ ^41^ ^]^ Single image acquisition was performed on a consistent area of each sample when possible. Videos in Figures V1‐V5 (Supporting Information) were stabilized to observe the deformation of consistent morphological features using Schneider's “Fix Translation and Batch Record Points” plugin for Fiji.^[^ ^42^ ^]^ Cyclic test data in Figures 8c and V10 (Supporting Information) was obtained at a lower resolution than other data, and was resized using Lanczos interpolation (Irfanview, Irfan Skiljan). Images for Figures 8b,c,V7,V9, and V10 (Supporting Information), due to the plane of focus, initially presented with PS appearing as a dark phase, PI appearing as a light phase, and out‐of‐plane deformations and cracks appearing black. This identification was confirmed by observing deformation behavior of the phases relative to each other, with the stiffer phase identified as PS. To maintain consistency in phase identification, these images’ lookup tables (LUTs) were inverted in Fiji, resulting in the appearance of light PS phases, dark PI phases, and white out‐of‐plane deformations and cracks.

## Conflict of Interest

The authors declare no conflict of interest.

## Author Contributions

All the authors contributed to the manuscript. The first two co‐authors contributed equally to the manuscript. The authors have approved the final version of the manuscript.

## Supporting information



Supporting Information

Supplemental Video 1

Supplemental Video 2

Supplemental Video 3

Supplemental Video 4

Supplemental Video 5

Supplemental Video 6

Supplemental Video 7

Supplemental Video 8

Supplemental Video 9

Supplemental Video 10

## Data Availability

The data supporting the findings of this study are available from the corresponding authors upon reasonable request.
