# Supplementary material for: Mechanical Deformation Behavior of Polymer Blend Thin Films
Source: Macromol Rapid Commun. 2024 Dec 31;46(4):2400736. doi: 10.1002/marc.202400736 (PMC11841662; doi:10.1002/marc.202400736)
Supplement: Supplementary file 1 — Supporting Information [file MARC-46-2400736-s011.docx]

Supporting information

**Mechanical Deformation Behavior of Polymer Blend Thin Films**

Geeta Pokhrel^1^, Hyungyung Jo^1^, Nicholas Christ^1^, Hyeyoung Son^1^_,_ John Howarter^1,2*^, and Chelsea Davis^1,3*^

G. Pokhrel, H. Jo, N.M. Christ, H. Son, J.A. Howarter, C.S. Davis

School of Materials Engineering, Purdue University, West Lafayette, IN, 47907, USA

J.A. Howarter

Environmental and Ecological Engineering, Purdue University, West Lafayette, IN, 47907, USA

C.S. Davis

Department of Mechanical Engineering, University of Delaware, Newark, DE, 19716, USA
C.S. Davis

Department of Materials Science and Engineering, University of Delaware, Newark, DE, 19716, USA

E-mail: ChelseaD@UDel.edu, howarter@purdue.edu


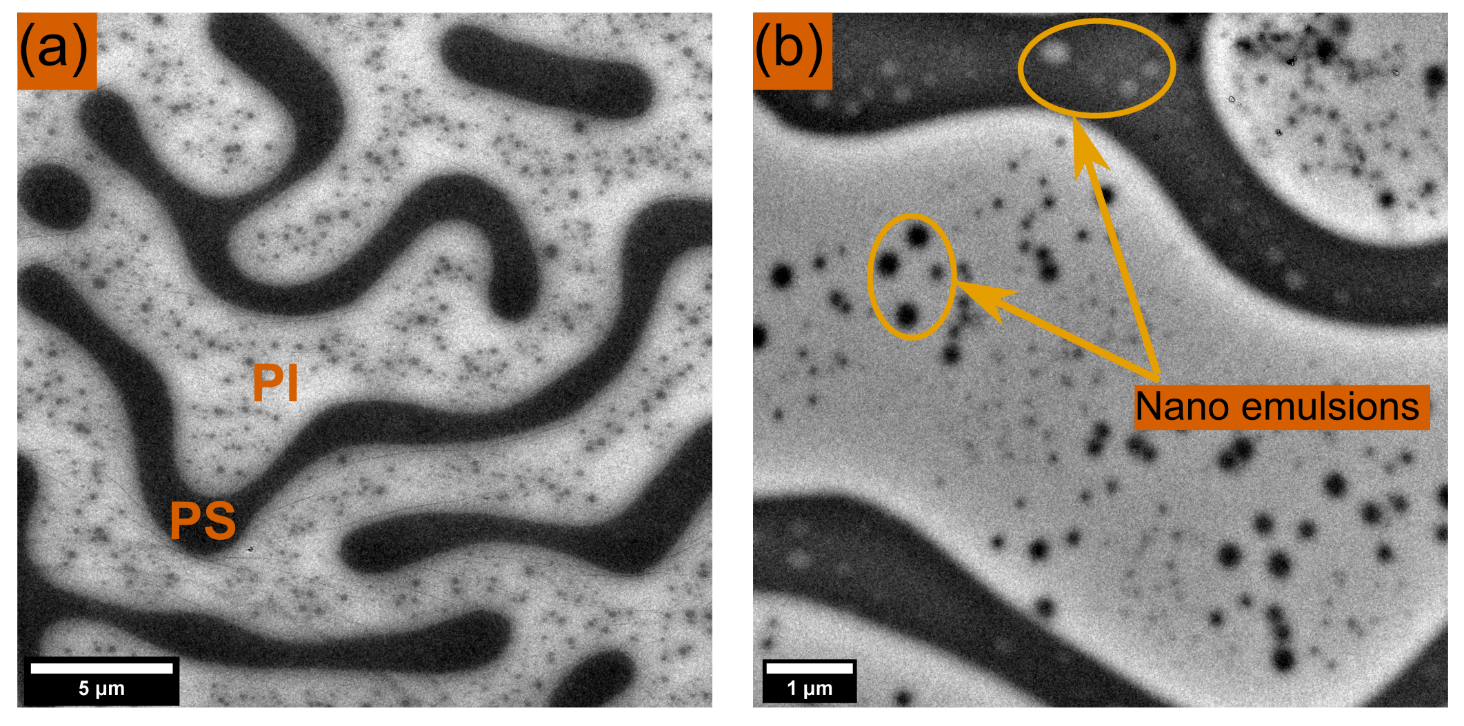


**Figure S1.** Transmission electron microscope (TEM) images showing (a) immiscible PS and PI polymeric phases represented by dark and light regions, respectively. (b) Representative nano emulsions of dark PS phases in lighter PI and of lighter PI phases in darker PS are shown inside the gold rings, which are not visible with optical microscope. The thin film formulation is 4.5PS and 5.5PI.

Areal fractions of the nanoemulsions were calculated for the image in (b) using image analysis software (Fiji, ImageJ, National Institutes of Health). ^[1]^ Nanoemulsions of PI comprised an areal fraction of 14.0% in the PS majority phases. PS comprised an areal fraction of 6.7% in the PI majority phases.


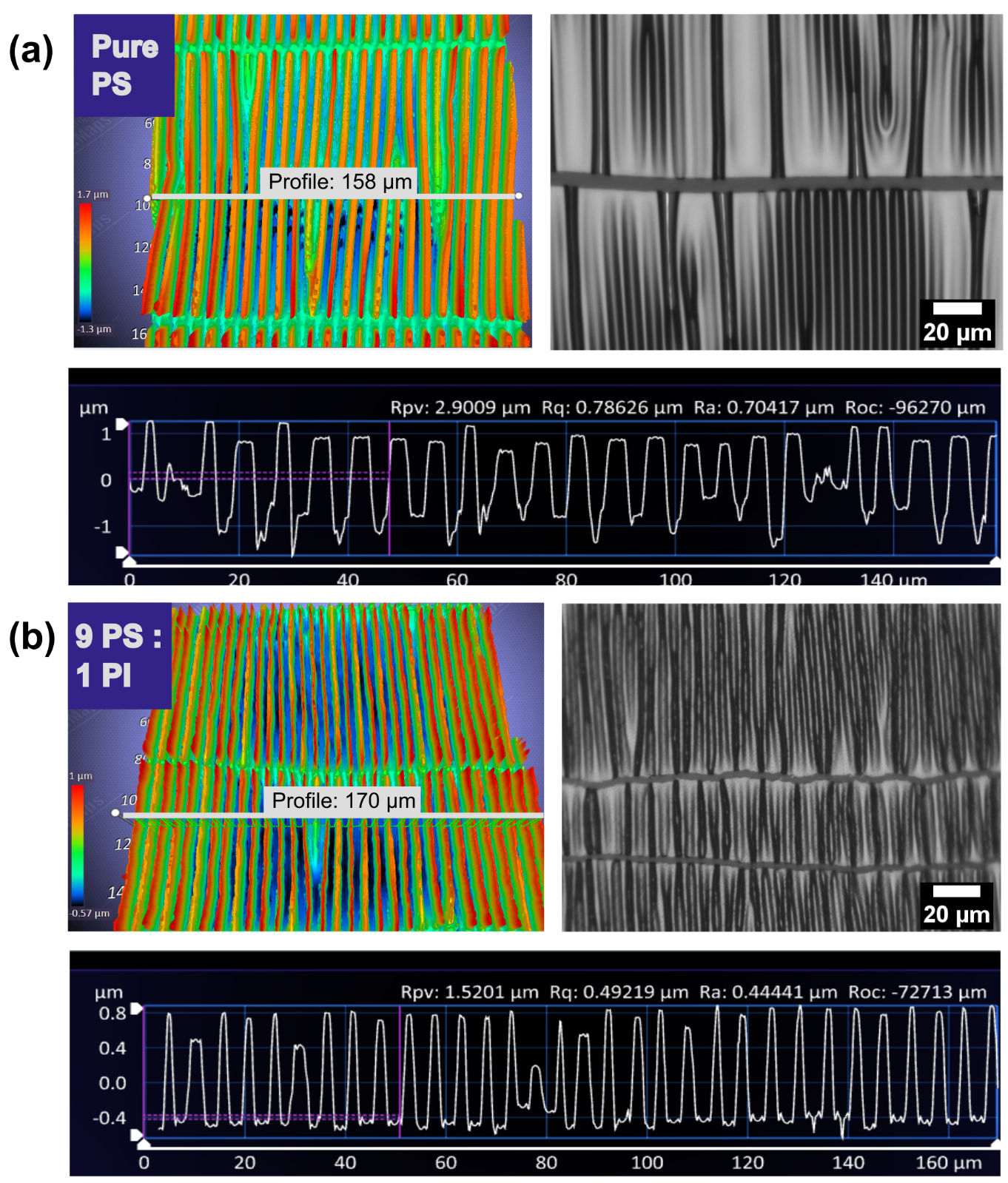


**Figure S2.** Optical profilometer (OP) and optical microscope images showing the deformation of thin films at 10% compression strain for (a) pure PS and (b) 9:1 ratio of PS and PI. Bottom images in (a) and (b) show the profile line graphs of pure PS and 9:1 PS/PI thin films at 10% compression taken using OP. The grey line in the OP image indicates section chosen for the profile line graph, with dimensions provided. Deformation varies between OP and optical microscope images due to different testing methods: stepwise test for the OP and quasi-continuous test for the optical microscope.

**Discussion on out-of-plane instabilities for pure PS and 9:1 ratio of PS/PI observed under optical profilometer and optical microscope:**

Various buckling instability modes, such as wrinkling, creasing, folding, and delaminated buckling, can be observed on thin films, depending on the modulus mismatch ratio of the film and substrate, the thickness of the thin film, the adhesion at the film-substrate interface, and the applied compressive strain.^[2,3]^ Across different mechanical tests i.e., stepwise or quasi-continuous and conditions i.e., tension, compression, and cyclic, several different deformation behaviors were observed including wrinkling, delamination, folds, and localized out-of-plane deformations.

The difference in stepwise tests for OP and quasi-continuous tests for optical microscopy have resulted in some discrepancies in the observed deformations particularly for pure PS and 9:1 PS/PI thin films. In a stepwise test, as shown in Figure 3 in main manuscript, wrinkle dislocations have time to propagate and annihilate with opposite dislocations as a mode of stress dissipation. In a quasi-continuous test, such as Figure 6, it is more likely that similar dislocations may collide, forming a high stress concentration that can lead to folding or delamination. For a pure PS in a quasi-continuous compression test, delamination propagation was continuously observed after reaching 10% strain since a residual stress remained on the film-substrate. As delamination of the PS film propagated, the PDMS substrate recovered to the initial flat state, and the stored strain energy on the adjacent wrinkles were released. For the 9:1 PS/PI blend thin film in a quasi-continuous compression test, wrinkling transitioned to folding because the modulus mismatch decreased, while other parameters, the thickness and the adhesion of films, were similarly given in this system.^[2]^

**
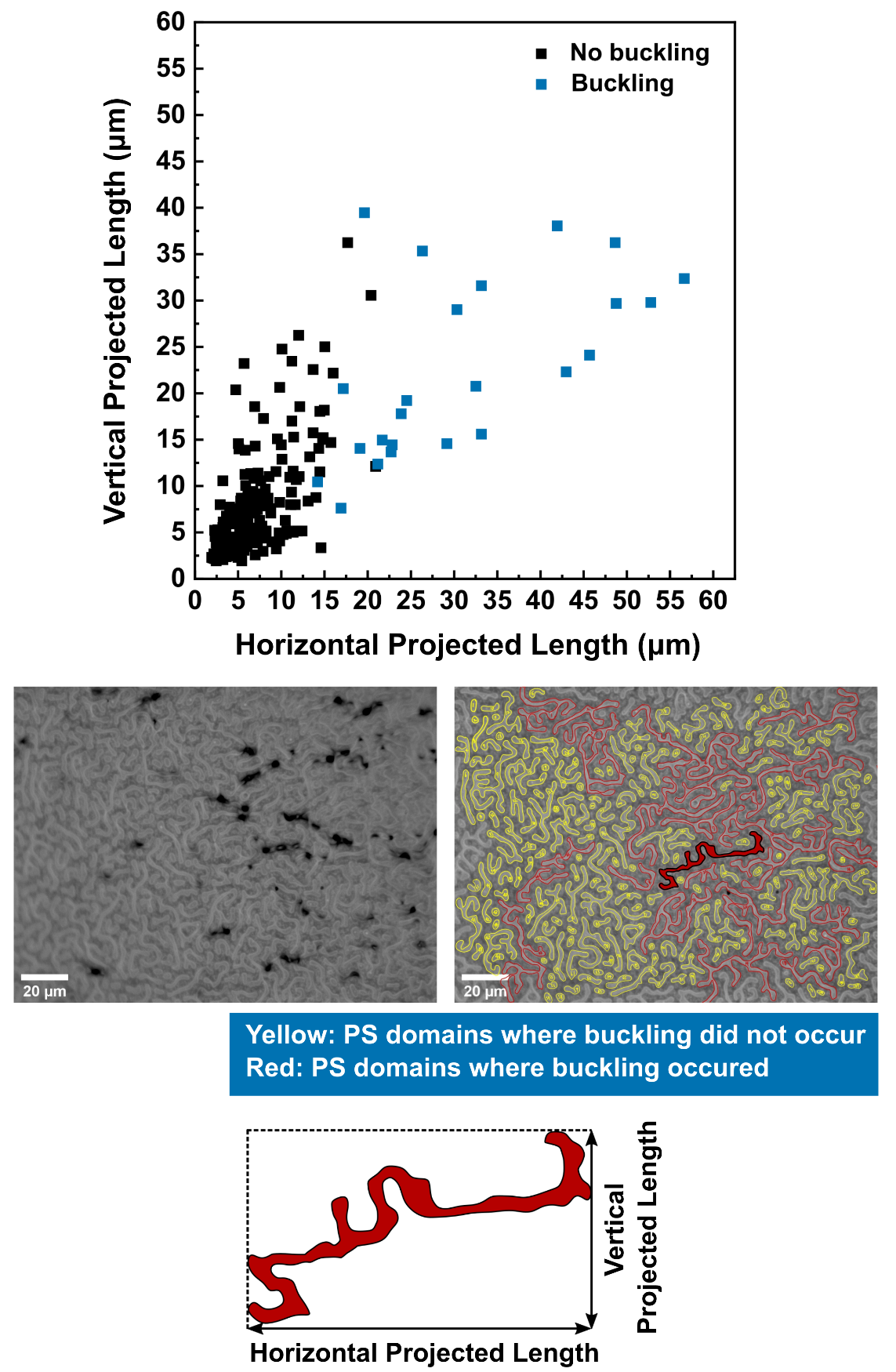
**

**Figure S3.** Quantitative analysis of PS domains that buckle and do not buckle during 10% compression. The average projected vertical and horizontal domains length in µm are represented in the graph.

**

**

**Figure S4.** Quantitative analysis of crack number density for all PS/PI polymer blend thin films after 100 cycles of 5% tension and compression. The crack density represents only the number of cracks, not their length. Localized cracks are defined as spanning the PS domain width and terminating in PI on either side.

**References:**

[1] J. Schindelin, I. Arganda-Carreras, E. Frise, V. Kaynig, M. Longair, T. Pietzsch, S. Preibisch, C. Rueden, S. Saalfeld, B. Schmid, J.-Y. Tinevez, D. J. White, V. Hartenstein, K. Eliceiri, P. Tomancak, A. Cardona, *Nat. Methods* **2012**, *9*, 676.

[2] Q. Wang, X. Zhao, Q. Wang, X. Zhao, *Sci. Rep.* **2015**, *5*, 1.

[3] Y. Ebata, A. B. Croll, A. J. Crosby, Yuri Ebata, A. B. Croll, A. J. Crosby, *Soft Matter* **2012**, *8*, 35.
